# Prior degree and academic performance in medical school: evidence for prioritising health students and moving away from a bio-medical science-focused entry stream

**DOI:** 10.1186/s12909-022-03768-y

**Published:** 2022-10-04

**Authors:** Kathryn Aston-Mourney, Janet McLeod, Leni R. Rivera, Bryony A. McNeill, Deborah L. Baldi

**Affiliations:** 1 School of Medicine, Deakin University, Geelong, Australia; 2 Deakin University, IMPACT, Institute for Innovation in Physical and Mental Health and Clinical Translation, Geelong, Australia

**Keywords:** Academic performance, Graduate entry medicine, Prior degree, Selection criteria

## Abstract

**Background:**

Given the importance of the selection process, many medical schools are reviewing their selection criteria. The traditional pathway for post-graduate medicine has been from science-based undergraduate degrees, however some programs are expanding their criteria. In this study we investigated academic success across all years and themes of the Deakin University medical degree, based on the type of degree undertaken prior to admission. We evaluated whether the traditional pathway of biomedical science into medicine should remain the undergraduate degree of choice, or whether other disciplines should be encouraged.

**Methods:**

Data from 1159 students entering the degree from 2008 to 2016 was collected including undergraduate degree, grade point average (GPA), Graduate Medical Schools Admission Test (GAMSAT) score and academic outcomes during the 4 years of the degree. Z-scores were calculated for each assessment within each cohort and analysed using a one sample t-test to determine if they differed from the cohort average. Z-scores between groups were analysed by 1-way ANOVA with LSD post-hoc analysis correcting for multiple comparisons.

**Results:**

The majority of students had Science (34.3%) or Biomedical Science (31.0%) backgrounds. 27.9% of students had a Health-related undergraduate degree with smaller numbers of students from Business (3.5%) and Humanities (3.4%) backgrounds. At entry, GPA and GAMSAT scores varied significantly with Biomedical Science and Science students having significantly higher scores than Health students. Health students consistently outperformed students from other disciplines in all themes while Biomedical Science students underperformed.

**Conclusions:**

Our data suggest that a Health-related undergraduate degree results in the best performance throughout medical school, whereas a Biomedical Science background is associated with lower performance. These findings challenge the traditional Biomedical Science pathway into medicine and suggest that a health background might be more favourable when determining the selection criteria for graduate entry into medicine.

## Background

Student selection is one of the most important responsibilities of medical education providers, as the decisions made at this point will ultimately determine the makeup of the medical workforce. As such, it is critical that the selection criteria are best able to identify those applicants who possess the necessary attributes to complete their studies and become successful practitioners. The role of a traditional premedical science-based degree prior to entry into medical school has long been debated [1]. There are many that would argue the idea of a defined premedical curriculum, where biology, chemistry, mathematics and physics are studied, to provide students with a sound scientific basis upon which to build their medical education, is a critical selection requirement.

The changing nature of medical education, particularly with the introduction of graduate entry programs, has challenged this paradigm with many schools recognising the effect of undergraduate education on the experience of the individual student through their medical degree [2–5]. Arguments for the inclusion of varied students in the medical curriculum note that the environment in which students learn and how they learn may be more important than what they learn [1]. Furthermore, the value added from having a diverse range of student backgrounds, bringing maturity, different life experience and perspectives [6], may augment the overall learning experience for the whole cohort [7]. In addition, students from non-science backgrounds have shown higher performance in behavioural science [8–11] and communication and interpersonal skills [12] and an increased likelihood of receiving prizes and awards [13].

Some programs have identified a background in non-science education is advantageous [5, 12, 14, 15], however some show non-science background students have higher numbers of failures in basic science [13], lower performance in physiology and biochemistry and increased attrition rates [8, 16]. In contrast, there are also studies which suggest there is little difference in academic success between medical students with a traditional science background than from those with previous studies in the fields of health and humanities and social sciences [5, 8, 9, 14–17].

Throughout this literature the emphasis has almost exclusively been on overall final outcomes of the medical degree. Given that students with limited exposure to the sciences prior to entering medical studies are perhaps more likely to encounter difficultly early in the degree, when the basic science of medicine is first taught, it seems that this is the stage at which some attention needs to be given to the progress of these students. In fact, the only study that did include an early measurement of progress showed that while there may not be differences in performance in the final years there was an increase in failure rate in year 1 and year 2 of the degree for students from a non-science background [13].

Given the importance of the selection process, many medical schools in Australasia and worldwide are reviewing their selection criteria. In order to make an evidence-based decision, more information is needed regarding the importance of a science background as a determinant of success in medical school. Furthermore, as most of the existing data stem from Northern Hemisphere studies, data from Australasian universities will be important for informing decision-making in this region.

Many studies have investigated whether the selection criteria used by medical schools are valid predictors of future success [16, 18–23], particularly academic performance. The aim of our study was to determine whether the type of degree undertaken prior to admission to graduate entry medicine also influences academic success across different years and areas of study. In particular, we sought to determine whether the traditional pathway of biomedical science into medicine should remain the undergraduate degree of choice for prospective medical students, or whether other disciplines should be encouraged.

## Methods

### Setting

In Australia medicine can be studied as either undergraduate or post-graduate entry. The Deakin University Bachelor of Medicine Bachelor of Surgery (BMBS, or Doctor of Medicine from 2019) is well suited to investigate whether premedical science training provides the best pathway to success in post-graduate medicine. The BMBS had no specific pre-requisites, rather admission was based on Grade Point Average (GPA), Graduate Medical School Admissions Test (GAMSAT) score and an interview. As such, there were students from a wide spectrum of academic backgrounds. The first 2 years of the degree were delivered on campus, with a focus on pre-clinical/preparation for clinical learning, followed by 2 years immersed in the clinical learning environment. Further, throughout these years student learning was assessed across the following themes: medical science (MS), ethics law and professionalism (ELP), and clinical practice (CP) with a higher weighting on the MS theme during years 1 and 2.

### Study design

Data from cohorts of students entering the Deakin BMBS degree in 2008 through to 2016 (*n* = 1159) was obtained with all identifiable information removed. This data was de-identified and provided in an excel worksheet by the data custodian. Data, including performance at entry and academic outcomes during the 4 years of the BMBS degree, was analysed based on student’s undergraduate background degrees. This study design was considered by the Deakin University Human Research Ethics Committee (ethics #2016–069) and determined to be low risk and was exempted from ethics review requirements.

### Background classification

Undergraduate background degrees were classified into four categories; Health, Science, Humanities and Business. Given the high number of science background students these were then separated into Biomedical Science (Biomed, degrees with a biological medical focus) and Science (degrees without a medical focus) to create five main categories. Students from a Health background were analysed as a whole and also classified into five sub-categories for further analysis. Where students had multiple background degrees their background was designated using the following priority order: Health > Biomed > Science > Humanities > Business.

### Variables

The variables analysed include the students’ Grade Point Average (GPA) and Graduate Medical School Admissions Test (GAMSAT) score upon entry to the Deakin BMBS degree.

For entry to Australian Medical Schools GPA is calculated on a seven-point scale set by the Graduate Entry Medical School Admissions System (GEMSAS) to ensure comparability between students with degrees from any university. Students are also required to sit the GAMSAT, which is designed to assess an applicant’s capacity to undertake high-level intellectual studies in the medical field.

Student progress throughout the pre-clinical and clinical years was assessed for each theme (MS; ELP; CP), in each of the four pre-clinical semesters and in each of the clinical years, as well as for the Observed Structured Clinical Examination (OSCE) which the students complete at the conclusion of the pre-clinical years and each clinical year. Progress was measured as grades for each assessment within each theme and the final grade for each unit. From these z-scores were calculated within each cohort by: z score = (mark-mean)/standard deviation.

### Analysis

All analyses were performed using SPSS software with *p* < 0.05 being considered significant. Missing data, for example when a student did not complete the degree, were removed pairwise. Z-scores of each group were analysed to determine if they differed from the expected cohort average of 0 using a one sample t-test with test value = 0.0. Z-scores between groups were analysed by 1-way ANOVA with least significant difference (LSD) post-hoc analysis correcting for multiple comparisons. Data are presented as mean +/− standard error of the mean (SEM). Percentage in the top or bottom 10% of the cohort was analysed with a one sample t-test with the test value = 0.10.

## Results

### Background degrees

There was a wide range of undergraduate degrees upon entry to the Deakin BMBS program (Table 1). The most common background degrees were Science (34.3%) and Biomed (31.0%). A similar number of students had a Health-related undergraduate degree (27.9%). Smaller numbers of students came from Humanities (3.4%) and Business (3.5%,) backgrounds.

**Table 1:** Undergraduate degree categories of students in Deakin BMBS

Category	Degree			N (%)
		Sub-category	N (% of health)	
HEALTH	Physiotherapy; Chiropractic; Occupational Therapy; Rehabilitation	Chiro/Physio	97 (30.0%)	323 (27.9%)
	Radiography/Medical Imaging	Imaging	31 (9.6%)	
	Nursing; Midwifery; Paramedics	Nursing	82 (25.4%)	
	Clinical Optometry; Podiatry; International /Public Health; Nutrition & Dietetics; Speech Pathology; Dental Science;	Other	30 (9.3%)	
	Pharmacy	Pharmacy	83 (25.37%)	
MEDICAL SCIENCE	Biomedical Science; Medical Science; Neuroscience; Anatomy; Human Biology			359 (31.0%)
SCIENCE	Psychology (no clinical); Science; Applied Science; Forensic Science; Biochemistry; Biotechnology; Cell Biology; Environment; Food Science			397 (34.3%)
HUMANITIES	Professional Development; Media and Communications; Journalism; Arts; Communication; Photography; Education; Languages; History; Liberal Studies; Music; Dance; Film and Screen Media; Dramatic Art			39 (3.4%)
BUSINESS	Law; Commerce; Engineering; Technology; Business; Computing; Accounting			41 (3.5%)

### Performance at entry to BMBS

**Fig. 1:**
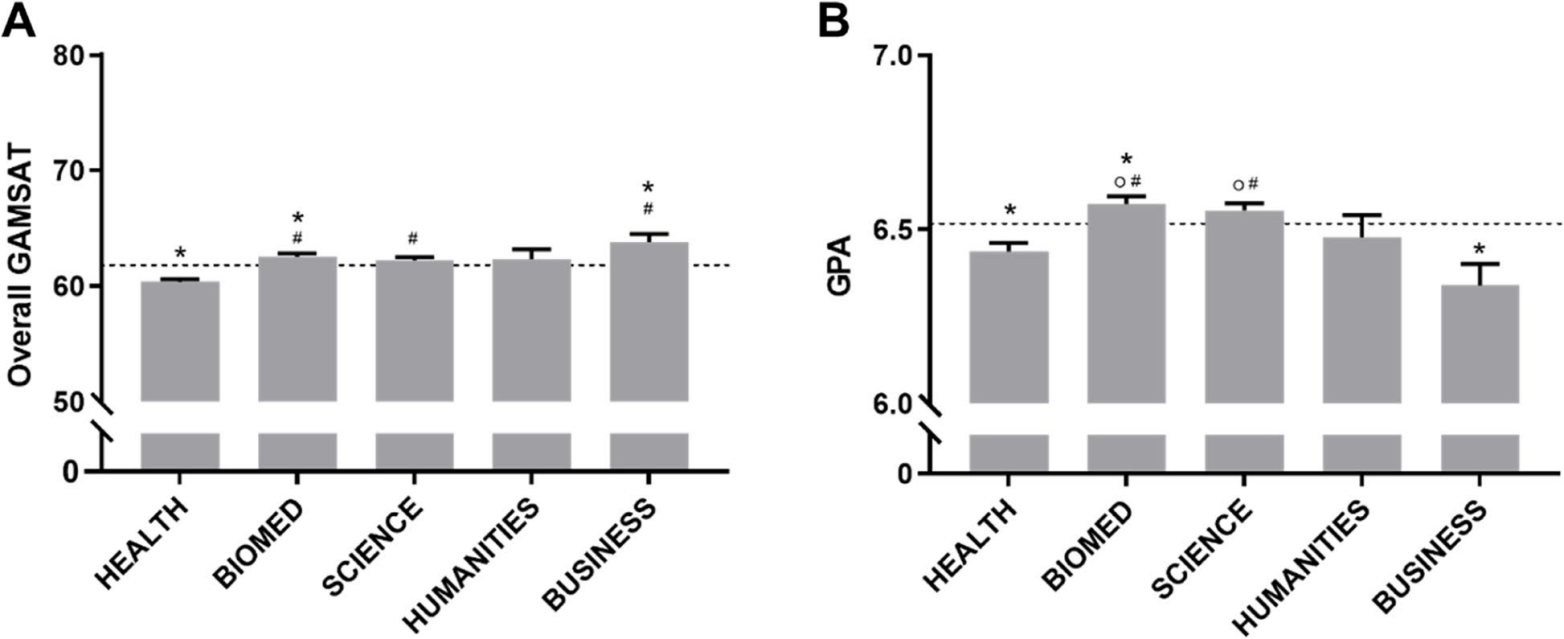
Entry Scores by Undergraduate Background. **A** GAMSAT Score and **B** GPA by Undergraduate Background. Dashed line represents average of entire cohort. *p < 0.05 vs. average, ^#^*p* < 0.05 vs. Health, ^0^*p* < 0.05 vs. Business

Both GAMSAT and GPA varied significantly between student backgrounds (Fig. 1). Compared to the cohort as a whole, students from a Business background had higher GAMSAT but lower GPA, Health students had lower GAMSAT and GPA while Biomed students had higher GAMSAT and GPA. Both Humanities and Science students were on par with the cohort as a whole for both scores. For both GAMSAT and GPA, Biomed and Science students had significantly higher scores than Health students.

### Performance in medical science

**Fig. 2:**
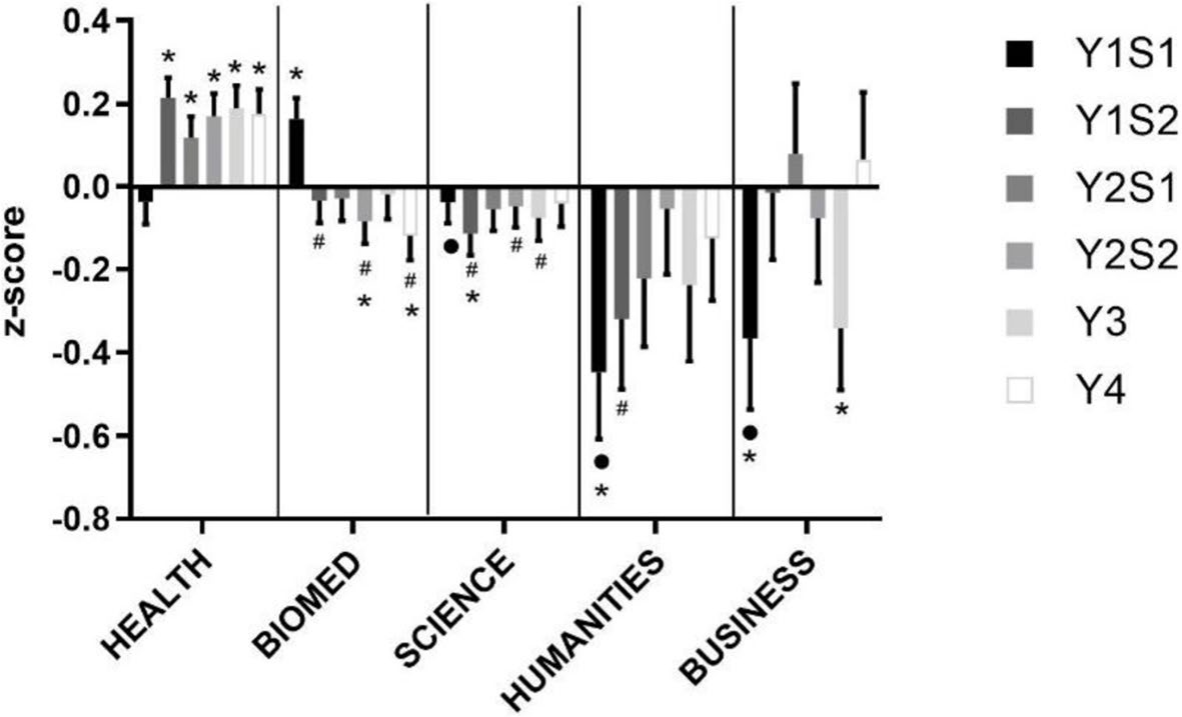
Medical Science Performance by Undergraduate Background. Performance by Undergraduate Background in the MS theme from year 1 to 4 (in increasing order, year (Y) semester (S)). *p < 0.05 vs. expected average of 0.0, ^•^*p* < 0.05 vs. Biomed, ^#^*p* < 0.05 vs. Health

Progress, as measured by grades during assessments, through MS varied by undergraduate background with a significant difference in performance in all four semesters of the pre-clinical years (Fig. 2). Business students’ performance was variable across the degree, but tended to be lower than average. Health students, while starting around average, performed significantly better than average after the first semester. Humanities students performed lower than average but tended to improve over time. Biomed students performed better than average only in the first semester of the degree and tended to perform slightly lower than average as the degree progressed. Science students tended to perform a little lower than average throughout the degree.

### Performance in ethics, law & Professionalism

**Fig. 3:**
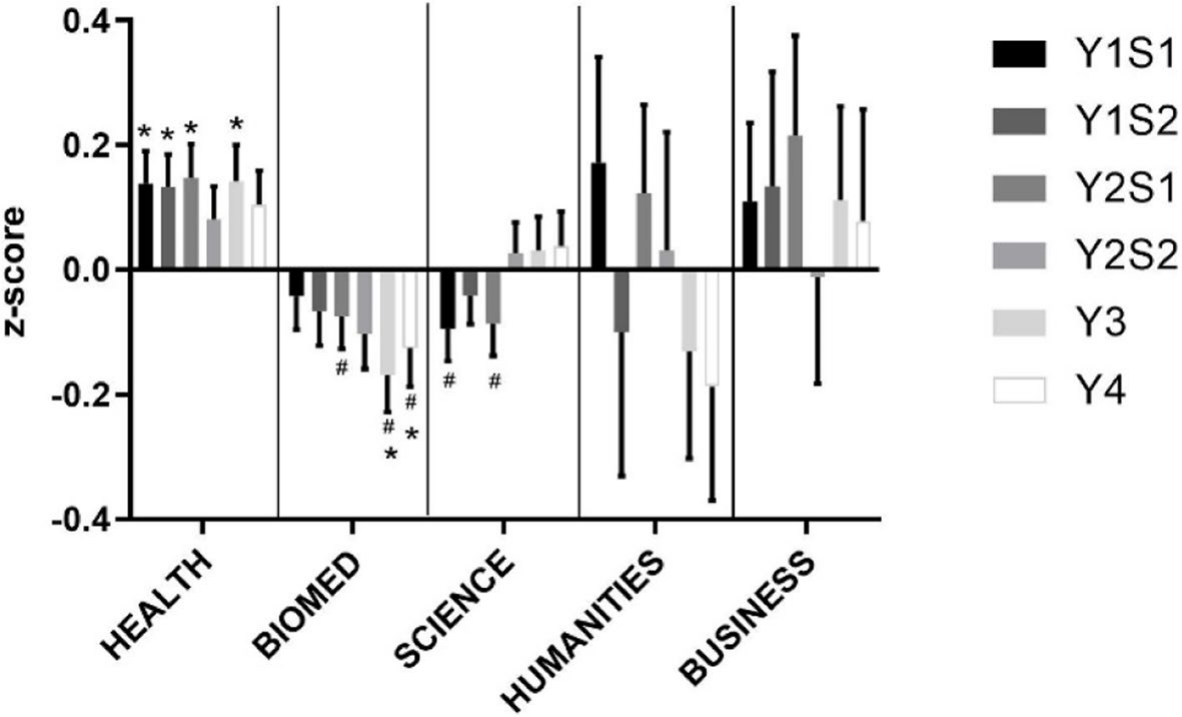
Ethics, Law and Professionalism Performance by Undergraduate Background. Performance by Undergraduate Background in the ELP theme from year 1 to 4 (in increasing order, year (Y) semester (S)). *p < 0.05 vs. expected average of 0.0, ^#^*p* < 0.05 vs. Health

Performance in ELP of students from different backgrounds also differed (Fig. 3). Health students were the only group that showed consistent and significantly higher than average performance. Business and Humanities students showed variable performance. Biomed students showed lower than average performance, especially in the later parts of the degree. Lastly Science students, while performing lower than Health students initially, tended to improve to average levels in the later parts of the degree.

### Performance in clinical practice

**Fig. 4:**
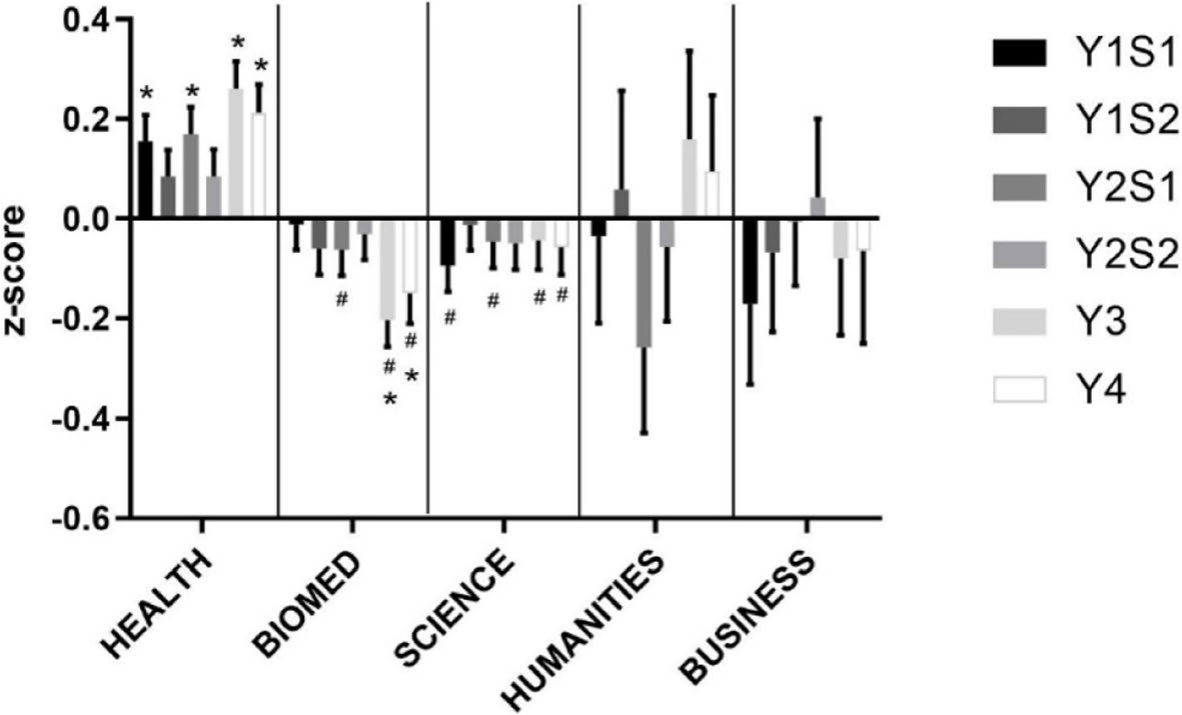
Clinical Practice Performance by Undergraduate Background. Performance by Undergraduate Background in the CP theme from year 1 to 4 (in increasing order, year (Y) semester (S)). *p < 0.05 vs. expected average of 0.0, ^#^*p* < 0.05 vs. Health

In the CP theme, Health students again consistently performed significantly higher than average (Fig. 4). Business and Humanities students showed variable performance. The performance of Biomed students started at average, but was subsequently lower than the other disciplines. Science students tended to perform slightly lower than average throughout the degree.

### Performance in observed structured clinical examinations

**Fig. 5:**
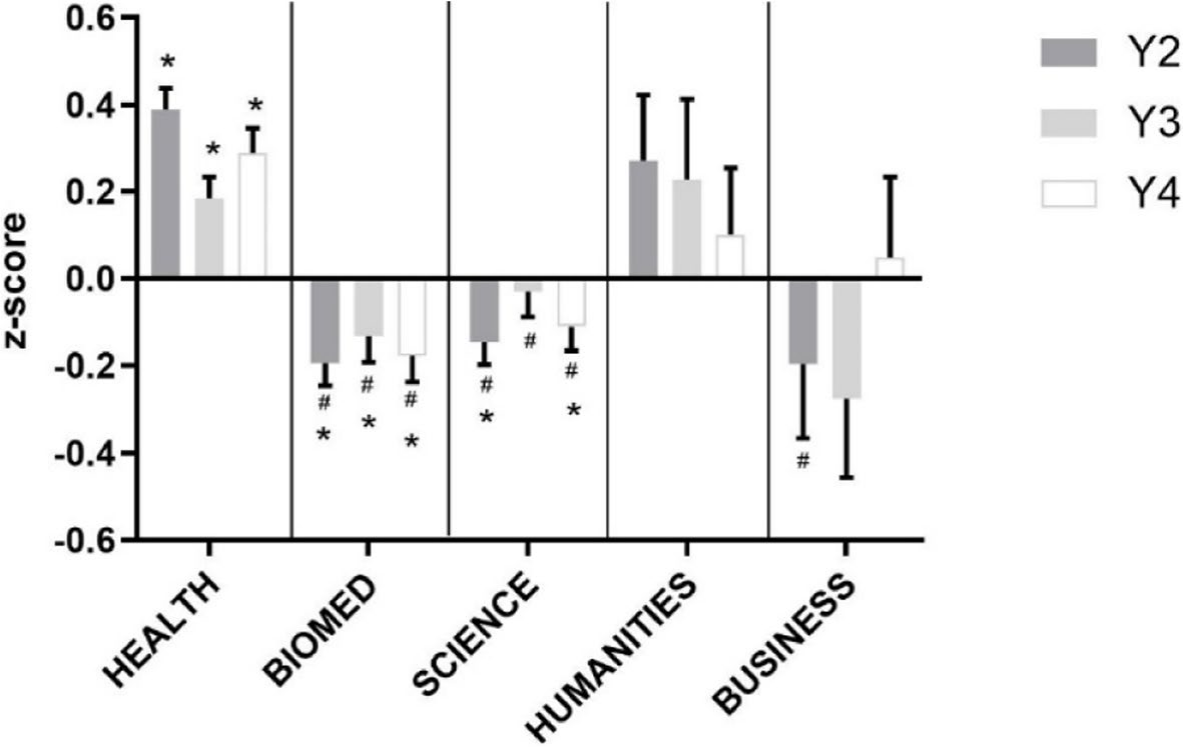
Observed Structured Clinical Examination Performance by Undergraduate Background. Performance by Undergraduate Background in the OSCE from year 1 to 4 (in increasing order, year (Y)). *p < 0.05 vs. expected average of 0.0, ^#^*p* < 0.05 vs. Health

The OSCE is widely used in Medical programs for assessing clinical competence, and is particularly valuable due to its high degree of reliability and validity [24]. OSCE performance was significantly different between undergraduate backgrounds (Fig. 5).

Specifically, Business students started off with lower z-scores compared with the other disciplines, but increased their performance to average in Year 4. Health students performed consistently better than average while Humanities students tended to perform better than average. Biomed and Science students performed lower than average throughout all 3 years of OSCEs.

### Overall performance

**Fig. 6:**
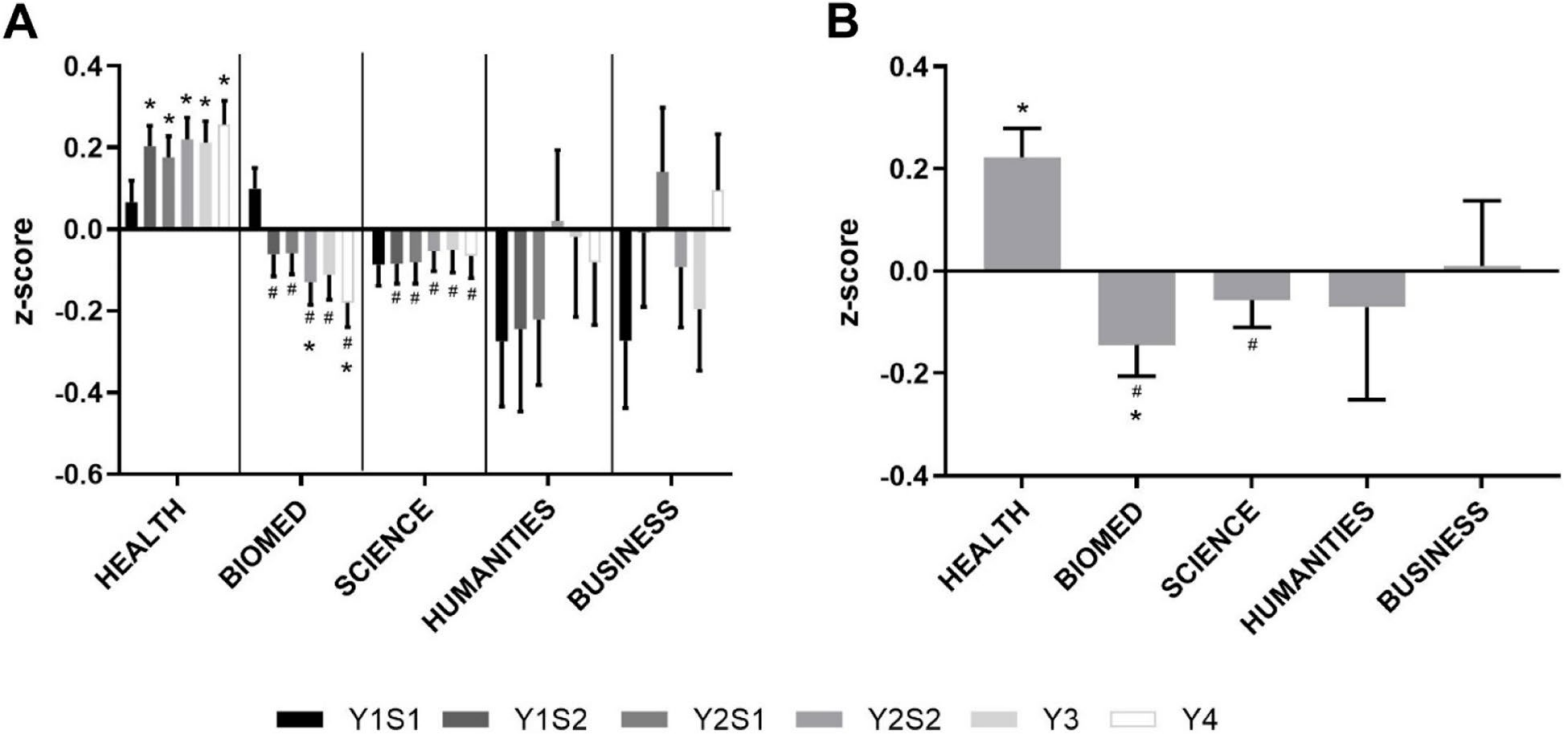
Overall Performance by Undergraduate Background. **A** Performance by Undergraduate Background overall for each semester/year from year 1 to 4 (in increasing order, year (Y) semester (S)) and **B** final grade for the degree (calculated from year 3 and 4). *p < 0.05 vs. expected average of 0.0, ^#^*p* < 0.05 vs. Health

Overall, for each unit/year and the final grade, Business students performed variably but evenly with the average of the cohort (Fig. 6). Health students performed consistently better. Humanities and Science students tended to perform lower than average, while Biomed students performed significantly lower than average.

**Fig. 7:**
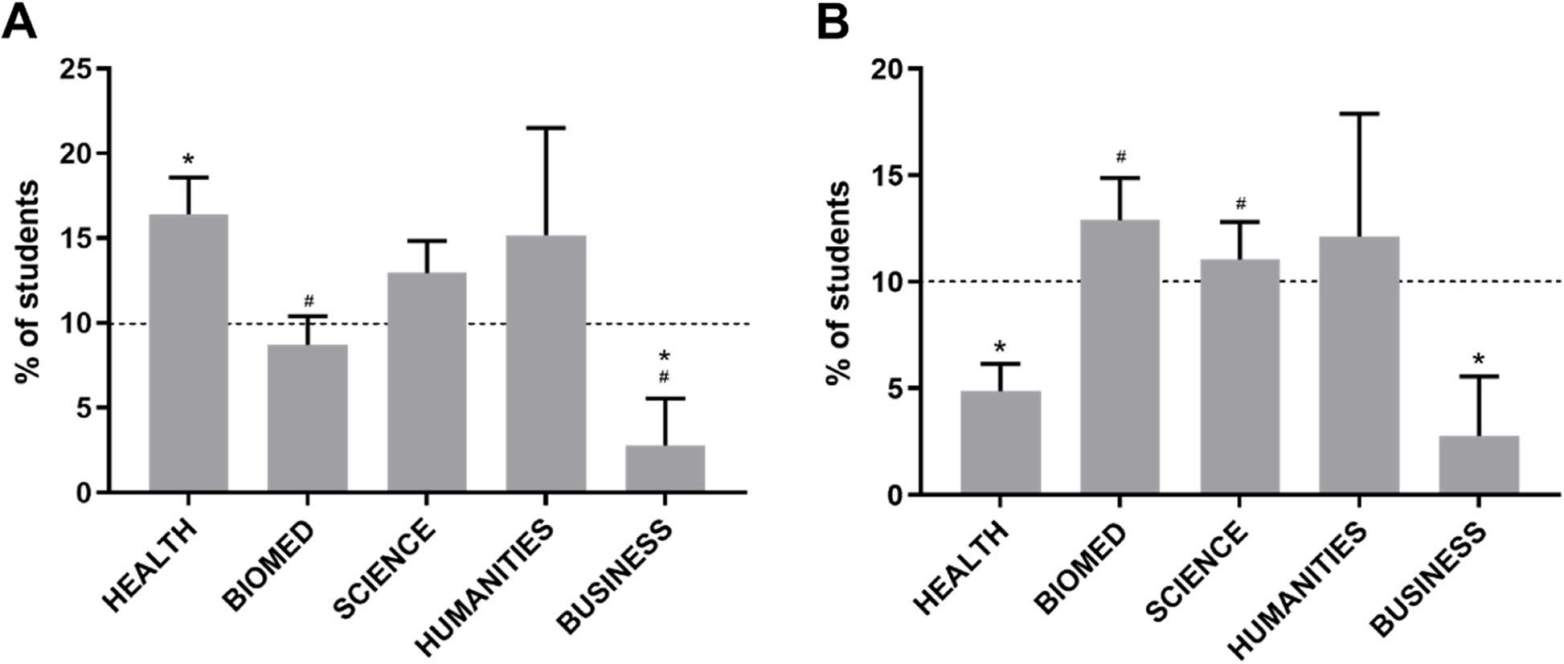
Top and Bottom Performing Students by Undergraduate Background. Percent of students by Undergraduate Background represented in the top (**A**) and bottom (**B**) 10% of students in final grade for the degree (calculated from year 3 and 4).*p < 0.05 vs. expected average of 10%, ^#^*p* < 0.05 vs. Health

Consistent with the previous measures of performance, Health students were significantly overrepresented in the top 10% performing students and underrepresented in the bottom 10% performing students (Fig. 7). Business students were underrepresented in both the top and bottom, while Humanities, Biomed and Science students were not significantly different to the expected average of 10%. Overall, Health students were represented significantly more in the top performing students, and less in the bottom performing students than Biomed students.

To determine whether progression rates through the degree differed by background degree, the proportion of students failing or discontinuing was determined. Failure rates differed by group (*p* = 0.002) with Health students showing the lowest fail rate (0.38%) and both Biomed and Science students (1 and 0.95% respectively) having significantly higher fail rates compared to Health (*p* < 0.001 and *p* = 0.001 respectively). The rate of discontinuation was not different amongst any of the groups.

### Health discipline analysis

As Health students were predominant in the highest performing group of students throughout the degree, we also sought to identify whether there was any difference between the specific Health disciplines. The background degrees of the 323 Health students were divided into 5 sub-categories as shown in Table 1.

**Fig. 8:**
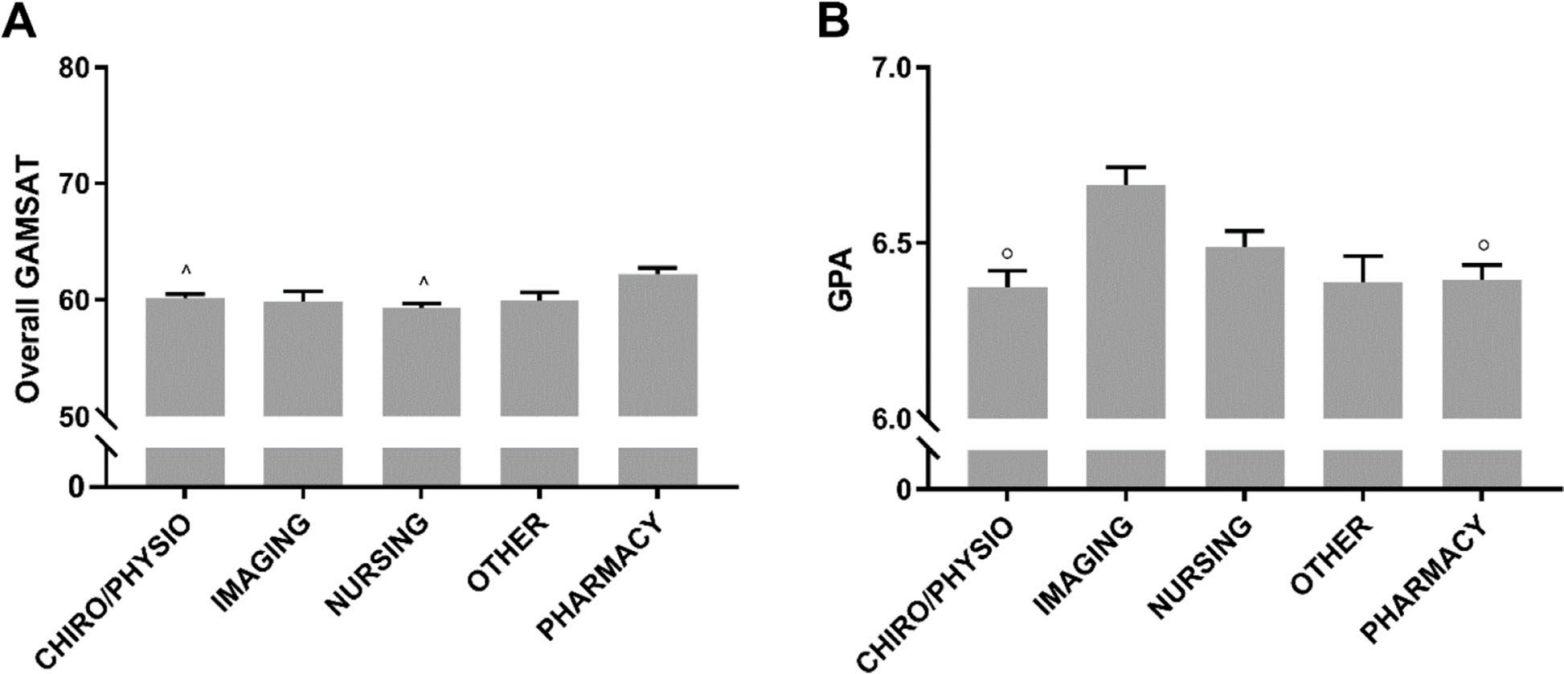
Entry Scores of Health Students by Discipline. **A** GAMSAT Score and **B** GPA of Health Students by Undergraduate Background. *p < 0.05 vs. average, ^^^*p* < 0.05 vs. Pharmacy, ^0^*p* < 0.05 vs. Imaging

At the point of entry into the course, Pharmacy students tended to have higher GAMSAT scores and Imaging students higher GPAs (Fig. 8A and B). There was no difference in the entry scores between any of the other Health disciplines.

**Fig. 9:**
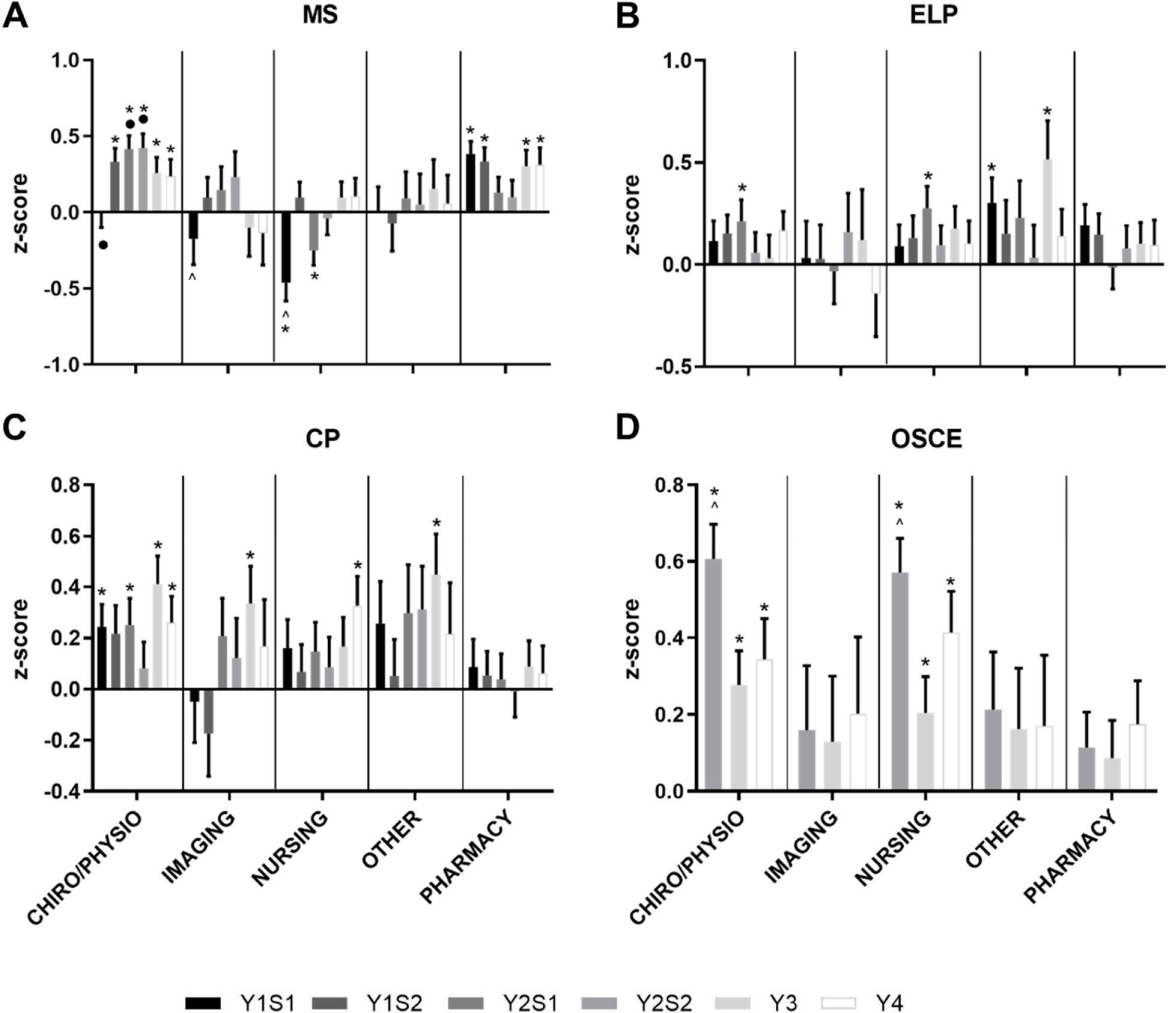
Academic Performance of Health Students by Discipline. Performance of Health students by Undergraduate Background in MS (**A**), ELP (**B**), CP (**C**) and OSCE (**D**) for each semester/year from year 1 to 4 (in increasing order, year (Y) semester (S)). *p < 0.05 vs. expected average of 0.0, ^•^*p* < 0.05 vs. Nursing, ^*p* < 0.05 vs. Pharmacy

During the degree (Fig. 9), Chiro/Physio students tended to perform better than average across all themes whilst Imaging students did not demonstrate increased performance in any theme. Nursing students showed higher performance in ELP, CP and OSCE but lower performance in MS in the first semester of the course, although this improved with time. Pharmacy students performed higher than average in MS but not different in the other themes. The remaining students (Other) showed some increased performance in ELP and CP.

**Fig. 10:**
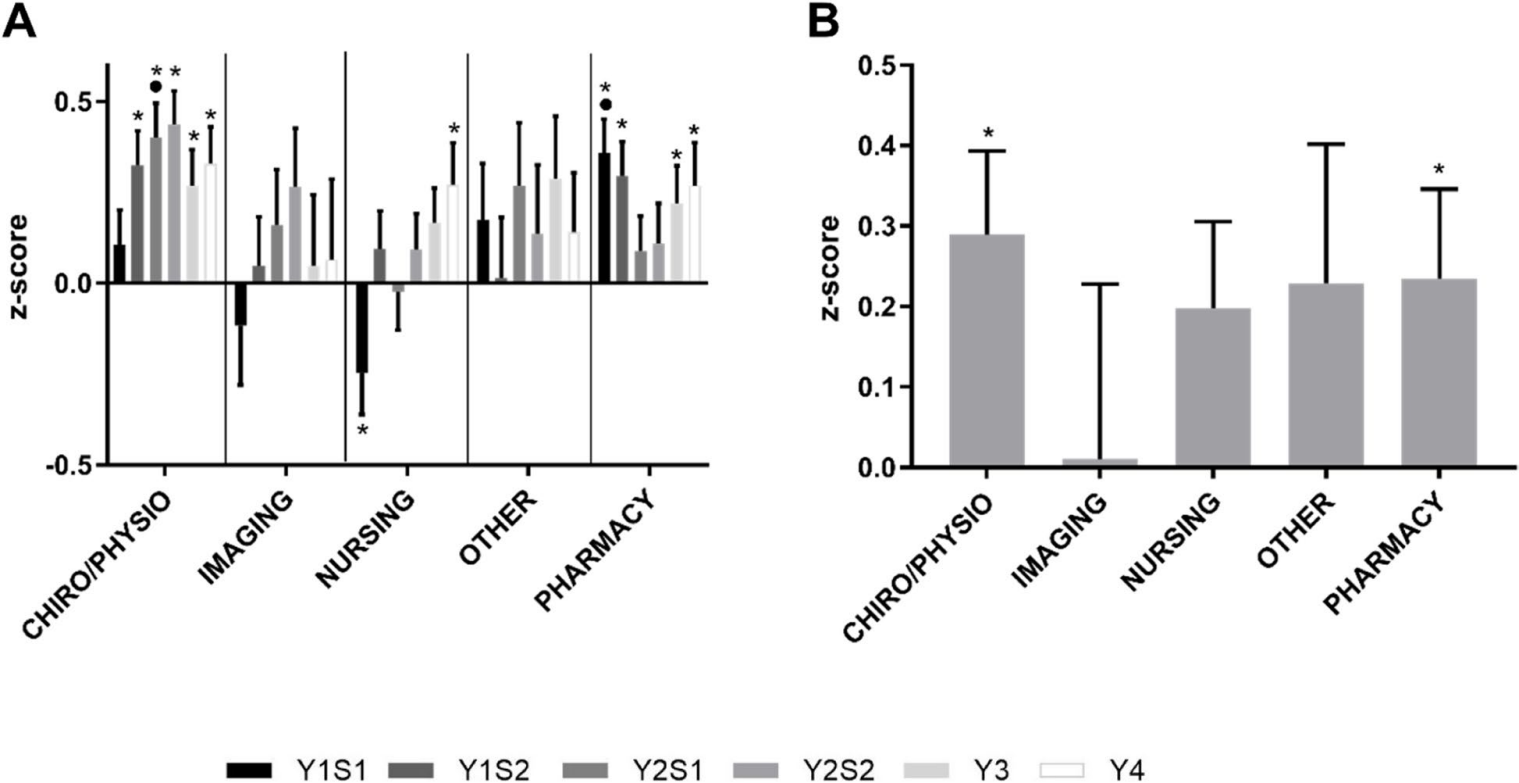
Overall Performance of Health Students by Discipline. **A** Performance of Health students by Undergraduate Background overall for each semester/year from year 1 to 4 (in increasing order, year (Y) semester (S)) and **B** final grade for the degree (calculated from year 3 and 4). *p < 0.05 vs. expected average of 0.0, ^•^*p* < 0.05 vs. Nursing

In terms of overall marks (Fig. 10), Imaging students were the only subgroup whose performance at the end of the degree was not higher than average, however there was very large variability within this group. Health students were significantly overrepresented in the top performing students and underrepresented in the lowest performing students, this tended to hold true for all subgroups, especially Chiro/Physio and Nursing, indicating all health disciplines performed very well. There was a very low fail and discontinue rate in all subgroups with no significant differences between groups (data not shown).

## Discussion

We aimed to determine whether the type of degree undertaken prior to admission to graduate entry medicine influences academic success across different years and themes of the degree. Our data suggest that a Health-related undergraduate degree results in the best performance at medical school. This challenges the traditional Biomedical Science pathway into medicine, and suggests that a health-related background should be viewed favourably when determining the selection criteria for entry into a medical degree.

Consistent with most other medical programs, a Biomedical or Science undergraduate degree was by far the most common pathway into the Deakin graduate BMBS degree. This suggests that these degrees are still viewed as the most appropriate pathway to a medical degree in Australia. However, while we note that these are very high performing students, as with all our cohort, these students tended to perform lower than the cohort on average. Collectively, these data suggest that a Biomedical degree is not necessarily the best pathway into medicine, and indeed those programs which stipulate a Biomedical pre-requisite degree are potentially missing out on high quality candidates who do not have this background.

A particular strength of the current study is that we were able to assess student performance across the different themes of the medical program, thus gaining insight into whether the various background degrees differentially prepare students for particular components of the course. Interestingly, the lower than average performance for Biomedical students occurred over all themes showing that there was no specific area of difficulty but an overall lower than average performance. In fact, the only point where Biomedical students showed significantly higher than average performance was in the first semester of year 1 in the medical science component of the course. This was not surprising given that the undergraduate Biomedical degree content is directly related to the content being taught and assessed in this semester. However, it was surprising that this trend did not hold to later semesters. For all following semesters, students with a Biomedical background performed on-par (two semesters) or significantly lower (three semesters) than average.

Given this clear result, the question remains as to why Biomedical and Science background students performed lower than the cohort average. At entry, the Biomedical students had slightly higher GPA and GAMSAT scores than the cohort average, while Science students were not different to the cohort average, indicating that these students have highly developed cognitive skills. It also stands to reason that the content being taught in these background degrees is relevant to, and would not detract from, the study of medicine. Recently a study showed that students from pre-medicine and medicine programs were significantly more likely to use deep learning and goal-oriented (rather than performance orientated) strategies compared with science students [25]. Furthermore, within the medical student cohort, there was no difference in the learning strategies between students with a science and non-science background, indicating that a propensity for deep learning is a common attribute of current and prospective medical students, and is unlikely to explain the differences observed in our study.

However, perhaps other important skills that are included in Health degrees are not included in Biomedical and Science degrees, or the degrees’ assessments do not adequately discriminate the students with the most appropriate skills for the study of medicine. For instance, if much of the assessment is based on knowledge recall, the highest performing students may be those with advanced rote learning skills rather than those with good clinical reasoning or problem-solving skills. Although GPA has previously been shown to be a good indicator of performance in medicine [5, 16], our finding that students with a Health background outperformed all other groups clearly highlights the limitations of using GPA as a primary determinant for entry into post-graduate medicine, and is an important outcome of the current study. Despite entering with lower GPA and GAMSAT scores, with the exception of first semester medical science, Health students performed higher in all themes for the duration of the degree, with significantly lower failure rates. These data are supported by previous reports from other Australian medical programs which also observed higher academic performance in students from a Health/Allied Health background compared with those from a biomedical science background, despite entering with relatively lower GPA [5, 16]. In this context, it is important to note that medical entrance tests and GPA scores may favour particular groups above others. Male gender, younger age, English as a primary language and higher socioeconomic status are all associated with higher performance in the GAMSAT [26, 27], resulting in an overrepresentation of these students in medical programs [28]. Further, students with a Health-related background degree have been reported to achieve lower GAMSAT scores than students from other backgrounds, with Biomedical and Science students having the highest scores [26], a finding consistent with our study. However, the results of our study clearly show that students from a Health-related background come into the medical degree with the most appropriate experience and skills to succeed in medicine. These data suggest that consideration of background degree in the selection process for post-graduate medical programs is warranted, and may provide a useful addition to the current selection tools, potentially offsetting some of their biases.

In our medical program, a Clinical Bonus is applied for applicants who hold a prior degree in an AHPRA (Australian Health Practitioner Regulation Agency) registered profession. Although there was some variability in student performance between the different Health categories, all Health disciplines (except imaging, which had very low numbers) tended to perform higher than the cohort average, especially in the later semesters. This indicates that all areas of allied health provide a suitable pathway into graduate-entry medicine and likely discriminate the students with the most appropriate strengths and skill sets for a career in medicine. Our results reinforce the use of the Health bonus, and indicate that it is appropriate for this bonus to be applied to applicants from all areas of Health, rather than restricted to a limited number of clinical qualifications. In the current study, we did not have access to additional information about the student population, however previous clinical experience, professional identity, age, and life experience may all contribute to the increased performance of Health students. Nonetheless, in applying a clinical bonus to students with a prior Health background, the particular strengths of these students are recognised, regardless of whether they are a direct result of their Heath studies or other factors.

Of note, in the current study, the failure and discontinuation rates for students from Business and Humanities degrees were not different from that of Health students, and indeed were lower than that of Biomed and Science students. These students comprised a very small percentage of our cohort (3.4 and 3.5% respectively) therefore we can only draw limited conclusions however, this indicates that students from these backgrounds are just as likely to successfully complete the degree as those students from Health or Science-related disciplines, and provides further support for the idea that a Biomedical pre-requisite for medicine is not necessary. Furthermore, increased diversity within the student cohort is highly beneficial for student learning, particularly in the group learning environment [5]. Clearly, more data from students with background degrees in Business and Humanities will be required to make any further conclusions regarding their preparedness for medicine and success following graduation.

A major strength of the current study is the large data pool, which includes results from over 1100 students across an 8-year period. However, there are also limitations to this data set. In particular, we did not have access to any information about our study participants other than their GPA, GAMSAT score, and previous degree. As such, we were not able to include factors such as age, socio-economic status, previous work experience, international/domestic student status, or any other health or disability factors that may contribute to differences in performance. It is possible that the differences reported here may reflect demographic differences between the study groups, rather than their background degree. Furthermore, due to the practical nature of Health degrees, students from these backgrounds will have spent some time working in clinical practice before commencing medicine, potentially contributing to their higher performance in the course. Nonetheless, in prioritising students from a Health background for acceptance into medical school, the particular strengths of these students are recognised, regardless of whether they are a direct result of their previous Health studies, or attributable to other factors.

## Conclusion

The best outcomes in our graduate medicine degree are achieved by students from a Health-related background rather than the traditional pre-medical science background. Therefore, we propose that applicants from a Health-related background should be promoted, and an appropriate loading applied to their application assessments. Furthermore, our findings suggest that Biomedical Science programs aimed at preparing students for post-graduate medicine may benefit from reviewing elements of their curriculum and assessment design to ensure that students are learning a range of skills and knowledge which will support their future success in medical school. In future studies, it will be important to extend these findings by investigating whether the differences in performance at medical school which we have identified here persist into the postgraduate environment.

## Data Availability

The raw data that support the findings of this study are not openly available due to it being individual human data. Processed data are available from the corresponding author upon reasonable request.
